# Facile Synthesis of Uniform Mesoporous Nb_2_O_5_ Micro-Flowers for Enhancing Photodegradation of Methyl Orange

**DOI:** 10.3390/ma14143783

**Published:** 2021-07-06

**Authors:** Jian-Ping Qiu, Huan-Qing Xie, Ya-Hao Wang, Lan Yu, Fang-Yuan Wang, Han-Song Chen, Zheng-Xin Fei, Chao-Qun Bian, Hui Mao, Jia-Biao Lian

**Affiliations:** 1 Xingzhi College, College of Geography and Environmental Sciences, College of Chemistry and Life Sciences, Zhejiang Normal University, Jinhua 321004, China; xhq970121@163.com (H.-Q.X.); yahaowang@zjnu.edu.cn (Y.-H.W.); fangyuanwang@zjnu.edu.com (F.-Y.W.); chenhs@zjnu.edu.cn (H.-S.C.); 2 College of Pharmaceutics, Jinhua Polytechnic, Jinhua 321007, China; haze_m@163.com (L.Y.); feizhengxin@jhc.edu.cn (Z.-X.F.); bian00@zju.edu.cn (C.-Q.B.); 3 Institute for Energy Research, Jiangsu University, Zhenjiang 212013, China

**Keywords:** photocatalysis, Nb_2_O_5_ micro-flowers, hydrothermal synthesis, methyl orange

## Abstract

The removal of organic pollutants using green environmental photocatalytic degradation techniques urgently need high-performance catalysts. In this work, a facile one-step hydrothermal technique has been successfully applied to synthesize a Nb_2_O_5_ photocatalyst with uniform micro-flower structure for the degradation of methyl orange (MO) under UV irradiation. These nanocatalysts are characterized by transmission and scanning electron microscopies (TEM and SEM), X-ray diffraction (XRD), Brunauer–Emmett–Teller (BET) method, and UV-Vis diffuse reflectance spectroscopy (DRS). It is found that the prepared Nb_2_O_5_ micro-flowers presents a good crystal phases and consist of 3D hierarchical nanosheets with 400–500 nm in diameter. The surface area is as large as 48.6 m^2^ g^−1^. Importantly, the Nb_2_O_5_ micro-flowers exhibit superior catalytic activity up to 99.9% for the photodegradation of MO within 20 mins, which is about 60-fold and 4-fold larger than that of without catalysts (W/O) and commercial TiO_2_ (P25) sample, respectively. This excellent performance may be attributed to 3D porous structure with abundant catalytic active sites.

## 1. Introduction

In recent years, the ever-increasing organic pollutants have caused serious threat to global environment [1,2,3]. Among different kinds of organic pollutants, azo dyes have been suspected to be human carcinogens as they can produce toxic aromatic amines. Methyl orange (MO), as one of the most used water-soluble azo dyes, have been extensively employed in painting, chemistry and papermaking, Unfortunately, the dye lost in the dyeing process are discharged into the water bodies, which can cause serious environmental issues and is harmful to human health. Therefore, ongoing interest in both the scientific and engineering communities is to find a pollution remediation strategy for degradation of organic pollutants into clean sources by selecting MO as a model organic pollutant [4,5,6,7]. 

Semiconductor photocatalysis technique has the outstanding characteristics of low cost, simple operation, efficient degradation of organic pollutants, and less secondary pollution. It has become an emerging environmental governance method in recent years [8,9,10]. Especially, the nanostructured semiconductor catalysts with high photocatalytic oxidation activity show a great potential application in organic pollutant degradation. Among the semiconductor-based photocatalysts, Niobium materials are promising materials as photocatalysts and solid acid catalysts, thus has been widely applied in different kinds of fields [11,12,13]. Because of the excellent properties like non-toxicity, corrosion resistance, high stability, and lots of surface acid sites, Niobium oxide (Nb_2_O_5_) has a wide range of catalytic activities for various reactions including dehydration, hydration, dehydrogenation, and many types of oxidation reactions [11,14,15,16,17,18]. 

Previous studies have indicated that morphologies of catalysts have a significant impact on its performance. Up to now, various interesting morphologies of Nb_2_O_5_ nanostructures, such as nanowires [11], nanobelts [19], nanorods [20], nanotubes [21], hollow nanospheres [22], nanoplates [23], have been synthesized. For instance, Zhao et al. fabricated Nb_2_O_5_ nanorods which have higher activity (99%) in the photodegradation of methylene blue than nanospheres (40%) attribute to the higher intensity of the 001 crystal plane after 90 min [24]. Du and coworkers found a rod-like Nb_2_O_5_ showed the best activity (95%) in the photocatalytic degradation of methylene blue after 150 min irradiation [25]. Qi et al. prepared Nb_2_O_5_ nanofibers via electrospun approach and prove the hexagonal-Nb_2_O_5_ nanofibers have a superior activity than orthorhombic -Nb_2_O_5_ during decomposing MO [26]. Therefore, many approaches have been proposed to control the morphology of Nb_2_O_5_, such as ionic liquid derived technology [25], sol–gel, precipitation, solvent thermal and hydrothermal methods. Among them, Hydrothermal method has advantages in crystallizing with a mild condition (<200 °C), and keeping the hydroxyl groups at the surfaces with a larger number of acid sites [27]. Thus, the hydrothermal route shows a high potential to produce Nb_2_O_5_ nanostructures with high photocatalytic activity, but has not been properly investigated

In this work, we have applied a facile hydrothermal approach for the fabrication of Nb_2_O_5_ with micro-flower. The microstructure and morphology, surface area, crystalline phase and optical property of as-obtained Nb_2_O_5_ catalyst has been systematically charactered by SEM and TEM, BET, XRD and UV/Vis spectrophotometer. Furthermore, its photocatalytic performance of degradation of MO under UV irradiation is investigated, and compared to commercial TiO_2_ (P25) and reported Nb_2_O_5_ catalyst. The mechanism for the superior performance of as-prepared Nb_2_O_5_ micro-flowers is discussed.

## 2. Materials and Methods

### 2.1. Preparation of Nb_2_O_5_

In the typical preparation method [15], niobium oxalate (1.5 mmol; Aladdin, 98%) and ammonium carbonate (7.5 mmol; Aladdin, 99.99%) were added to deionized water (30 mL). After stirring for 15 min, the mixture was transferred to a 50 mL polytetrafluoroethylene lined autoclave, sealed in an oven, heated to 200 °C and kept for 12 h. The white precipitate was collected, washed with ethanol and water. Finally, the samples were kept at 60 °C for 3 h in in a vacuum oven, and the Nb_2_O_5_ micro flower were obtained. 

### 2.2. Characterization Methods

An X-ray diffraction (XRD) analysis of the catalysts was carried out at the XPert Pro MPD (PANalytical B.V., Almelo, The Netherlands) with Cu-Kα radiation generated at 45 kV and 40 mA. The morphologies of the catalysts were charactered by means of field emission scanning electron microscopy (FESEM, S-4800; Hitachi, Tokyo, Japan), which was operated at an accelerating voltage of 4 kV. The transmission electron microscopy (TEM) investigation was performed at the Tecnai G2F30 S-Twin TEM (FEI, Amsterdam, The Netherlands). The X-ray photoelectron spectroscopy (XPS) experiments were performed with a Thermo Scientific ESCA-Lab-200i-XL spectrometer (Waltham, MA, USA), which utilized monochromatic Al Alα radiation (1486.6 eV), and the C 1s and N 1s peak spectra were analyzed by using XPS Peak 4.1 software. To quantify the specific surface areas of examined catalysts, Brunauer–Emmett–Teller analysis was performed (BET, ASAT 2020M+C, Micromeritics, Micromeritics Instrument Co., Norcross, GA, USA), using the nitrogen adsorption and desorption isotherms at liquid nitrogen temperature (77 K). UV-Vis diffuse reflectance spectroscopy was recorded on a UV/Vis spectrophotometer (UV/Vis DRS, UV-2550, Shimadzu, Tokyo, Japan) over the wavelength range between 200 and 800 nm.

### 2.3. Photocatalytic Activity Tests

The photocatalytic degradation of MO was carried out in a cylindrical reactor under UV irradiation. A thermostat (THD-2015, Ningbo Tianheng Instrument Factory, Ningbo, China) was employed to maintain the temperature of the reactant solution at 25 °C. For each test, 5 mg of ground photocatalyst powder was dispersed in 100 mL of an aqueous solution containing 20 mg L^−^^1^ MO. the samples were collected and analyzed at 463 nm of the UV/Vis spectrophotometer (Shimad-zu-1601pc) within the preset time interval. Before the photocatalytic degradation experiment, the dye samples were continuously stirred in the dark for about 15 min to eliminate the adsorption effect.

## 3. Results and Discussion

### 3.1. Characterization of Nb_2_O_5_ Micro-flowers

**Figure 1:**
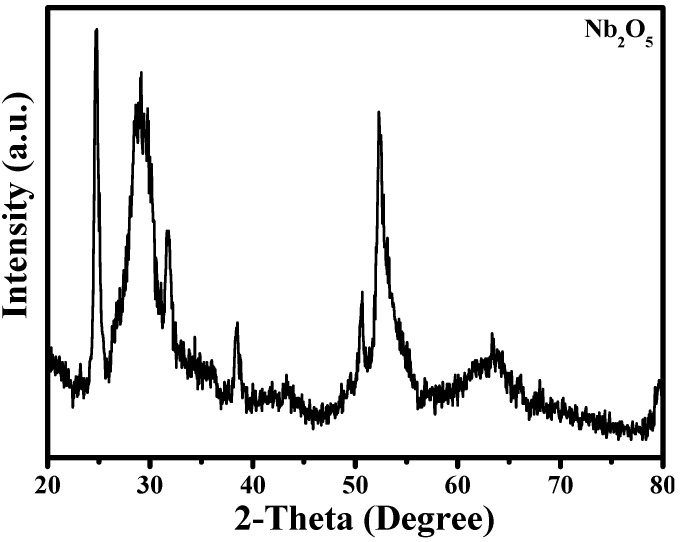
XRD patterns of the as-prepared Nb_2_O_5_ micro-flower.

The microstructure analysis of the as-obtained Nb_2_O_5_ micro-flower sample was carried out by using powder X-ray diffraction (XRD), as shown in the Figure 1. Similar to reported literatures, the main diffraction peak located at 24.8°, 29.9°, 32°, 38.9°, 44.3°, 51°, 52.6°, 53°, 64°, can be indexed to (121), (324), (314), (518), (809), (1010), (1119), (936), (154) plane of Nb_2_O_5_ (JCPDS 19-0862#) [21,22,23]. Moreover, the sharp peak indicates the high crystallinity of as-obtained Nb_2_O_5_ sample, which could enhance the photocatalytic degradation of organic molecules according to previous reports [15].

**Figure 2:**
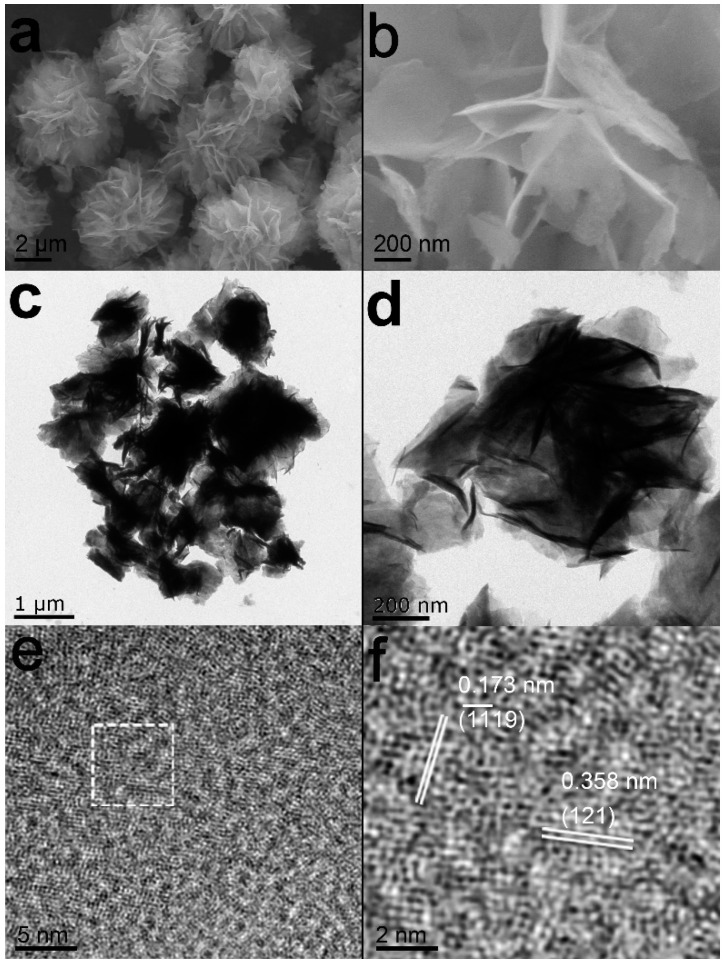
Low- and high magnification FE-SEM images (**a**,**b**), TEM images (**c**,**d**) and typical HRTEM images (**e**,**f**) of Nb_2_O_5_ micro-flower.

The morphology of as-obtained Nb_2_O_5_ sample was detected by using field emission scanning electron microscopy (FE-SEM), transmission electron microscopy (TEM), as well as high-resolution transmission electron microscope (HRTEM). Figure 2a reveals that the material has a structure consisting of uniform micro-flowers with a diameter of 2–3 micrometers. Each flower possesses a 3D structure composed of nanosheets as displayed in More structure details are further confirmed by TEM images as presented in Figure 2c. Indeed, the lateral sizes of these nanosheets are typically in the ranges of 400–500 nm in diameter (Figure 2d). Figure 2e,f present the HRTEM image of the sample, the lattice fringes in the magnified HRTEM image (Figure 2f) are found to be ca.0.358 and 0.173 nm, in accordance with the planes of the (121) and (1119) d spacings of Nb_2_O_5_ [15], respectively.

**Figure 3:**
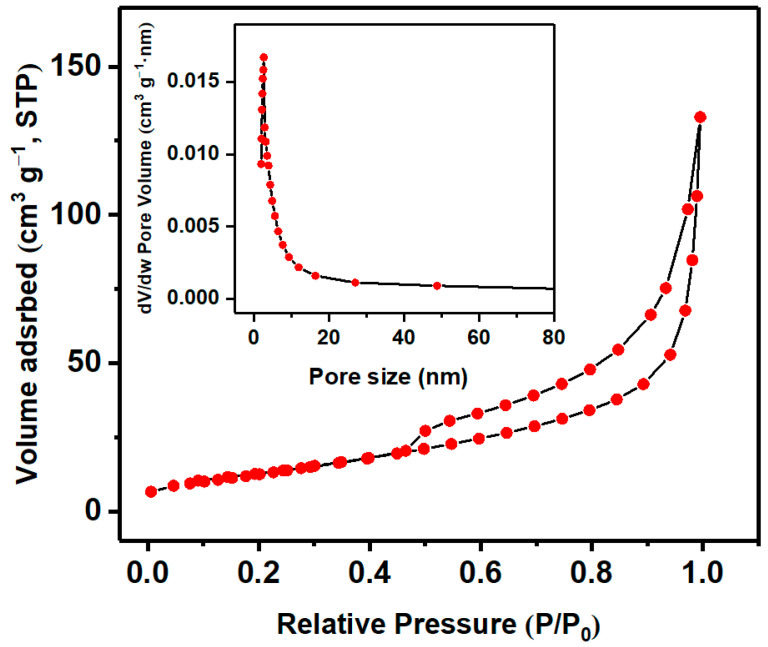
Nitrogen adsorption-desorption isotherm and BJH pore size distribution of Nb_2_O_5_.

To investigate the porosity and specific surface area of the as-obtained materials, the N_2_ adsorption-desorption measurement is conducted. As displayed in Figure 3, the Nb_2_O_5_ has type IV isotherm with a discrete hysteresis loop (H^3^ typedepecit) in the higher relative pressure (P/P_0_, 0.51–1.0) range, indicating the presence of slit-like pores and capillary condensation in the mesoporous. This mesopore feature could be further confirmed by the relevant pore size distributions as implied in the inset of Figure 3. From the plot, the surface area of Nb_2_O_5_ micro-flowers is estimated at 48.6 m^2^ g^−1^, which is larger than the reported Nb_2_O_5_ hollow fibers (32.8 m^2^ g^−1^) [28], hexagonal-like Nb_2_O_5_ (31.7 m^2^ g^−1^) [23] and Nb_2_O_5_ (N-100) (2.45 m^2^ g^−1^) [29]. This suggests that the hierarchical structure of Nb_2_O_5_ micro-flowers has a large surface area and accordingly provide more catalytically active sites during the process of molecular adsorption and reaction.

**Figure 4:**
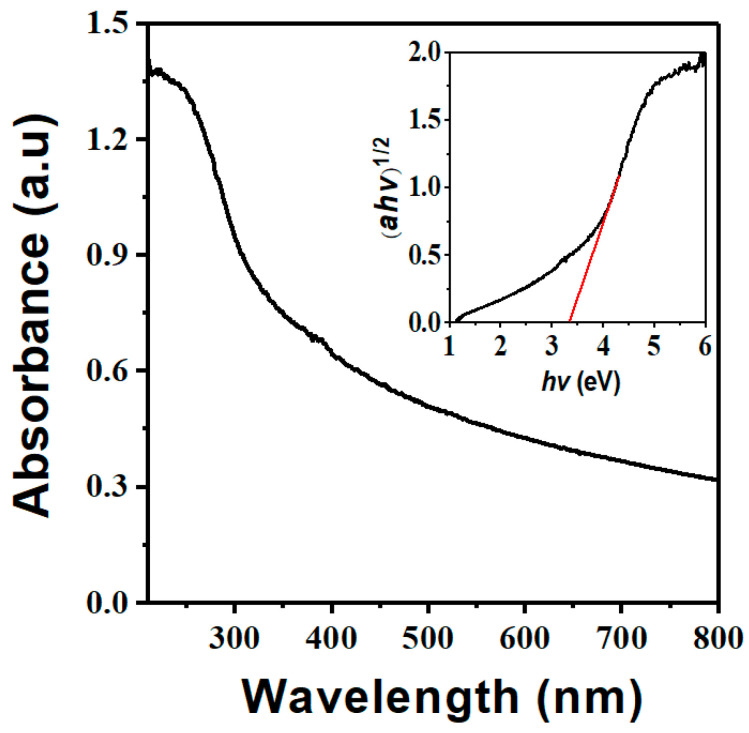
UV/Vis DRS and Tauc plots of of Nb_2_O_5_.

The optical absorption of Nb_2_O_5_ micro-flowers is investigated by UV/Vis diffuse reflectance spectroscopy (DRS) spectrum. As illustrated in Figure 4, the Nb_2_O_5_ shows a strong absorption bands in the range of 230–400 nm, and the absorption edge value of Nb_2_O_5_ is observed at 384 nm. This enhanced optical absorption would make the Nb_2_O_5_ effectively interact and employ incident light to generate abundant active species for driving catalytic reactions. Based on previously published literatures, the band gap of the as-prepared materials can be measured by the following equation [30,31]:

(1) $$\alpha h\nu \;=\;A{\left(h\nu \;-\;\mathrm{Eg}\right)}^{\frac{n}{2}}$$

where A is a constant, Eg is the band gap, and *n* is a number that varies with the transition of the semiconductor which takes value 4 for indirectly transition [30,31]. Therefore, as can be seen from the inset of Figure 4, the corresponding band gap value of Nb_2_O_5_ micro-flowers is estimated to be 3.34 eV similar to the reported values of 3.40 eV for Nb_2_O_5_ NC [32].

### 3.2. Photoactivity Evaluation of As-Prepared Materials

**Figure 5:**
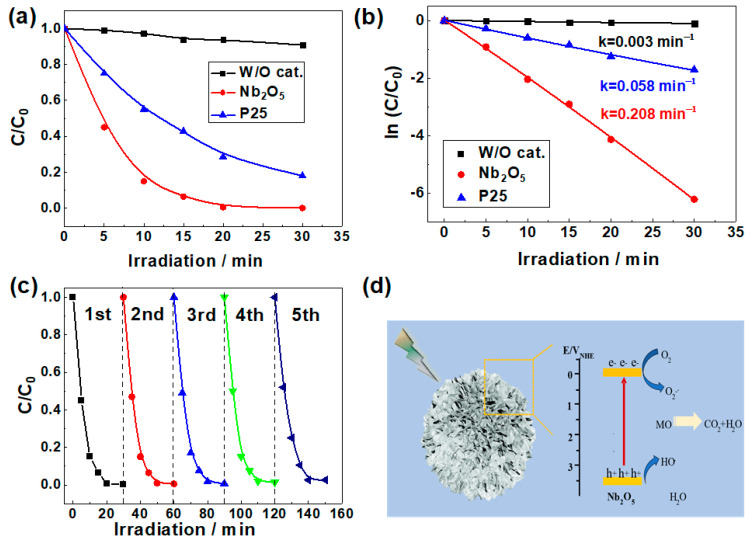
(**a**) Curves of MO dye photooxidation catalyzed by the as-prepared samples under UV irradiation; (**b**) pseudo first-order reaction kinetics of the as-prepared samples for degradation of MO; (**c**) Cycling performance of photocatalytic degradation of MO over Nb_2_O_5_ micro-flowers; (**d**) Photocatalytic degradation mechanism of MO on mesoporous Nb_2_O_5_ micro-flowers.

The photocatalytic activity is evaluated by the degradation of MO under UV irradiation. Figure 5a shows the photocatalytic performance of as-prepared catalysts. Obviously, the Nb_2_O_5_ micro-flowers completely bleach MO in only 20 min, at which the blank solution without catalysts and P25 sample show a photooxidation efficiency of 6.3% and 81.4%, respectively. Furthermore, the photocatalytic experimental data are found to fit pseudo-first-order kinetics as shown in Figure 5b, and the degradation constants for W/O sample, P25 catalyst and Nb_2_O_5_ micro-flowers are 0.003, 0.058, and 0.208 min^−1^, respectively. The apparent rate constant of Nb_2_O_5_ micro-flowers is almost 4 times higher than that of P25, revealing superior performance of Nb_2_O_5_ micro-flowers.

We also compare the photocatalytic performance of the present Nb_2_O_5_ micro-flowers with those of previously reported Nb_2_O_5_ nanostructures, the data are summarized in Table 1. Obviously, the Nb_2_O_5_ micro-flowers in the present work with highest efficiency up to 99.9% in the shortest time, is superior to other reported Nb_2_O_5_ catalysts for the photodegradation of dye wastewater. This might arise from that the large surface area of 3D porous structure, and greater number of acid sites generated by the hydrothermal method for photooxidation.

**Table 1:** A comparison of the photocatalytic performance.

Catalysts	Surface Area (m^2^ g^−1^)	Dye	Reaction Time (min)	Efficiency	Reference
Rod-like Nb_2_O_5_	/	Methylene Blue	150	95%	[25]
Nb_2_O_5_ (N-100)	2.445	Methylene Blue	270	55%	[29]
H-Nb_2_O_5_ hollow fibers	32.8	Methylene orange	50	93%	[28]
Sphere-like Nb_2_O_5_	104.3	Methylene blue	50	87%	[22]
		Rose bengal	180	62%	
Hexagonal-like Nb_2_O_5_ nanoplates	31.7	Methylene blue	60	92%	[23]
		Rhdamine B	60	98%	
Micro-flowers Nb_2_O_5_ (this work)	48.6	Methylene orange	20	99.9%	This work

Next, we also performance the reuse tests of the obtained Nb_2_O_5_ micro-flowers. Notably, after 5 cycles, the photo-catalytic performance only slightly decreased under UV light as shown in Figure 5c. This confirms the high stability of Nb_2_O_5_ micro-flowers. All the above results demonstrate that Nb_2_O_5_ micro-flowers is a good photocatalyst for degradation of MO.

To reveal the enhanced photocatalytic activity, a mechanism proposed for the photodegradation of methyl orange by Nb_2_O_5_ is illustrated in Figure 5d. As evidenced above, Nb_2_O_5_ micro-flowers had a well-developed mesoporous structure with a large surface area and adsorption capacitance, which provides a great number of active adsorption sites. An important step during the catalytic process is the adsorption of reacting substances onto the surface of the catalyst [33,34]. Then the photoelectron can be easily trapped by electronic acceptors like adsorbed O_2_, to further produce a superoxide radical anion (O_2_^−^), whereas the photo-induced holes can be easily trapped by organic pollutants, to further oxidize organic pollutants [35,36].

Furthermore, the two sides of the pore wall individually provide a place for the oxidation and reduction reaction, which facilitate separation of the electrons and holes. This might be the most beneficial character of mesoporous Nb_2_O_5_ to work as a high-performance photocatalyst.

## 4. Conclusions

In conclusion, we have developed a facile hydrothermal process to fabricate uniform micro-flower Nb_2_O_5_ photocatalysts for degrading MO under UV irradiation. The morphology and structures characterizations show that the prepared Nb_2_O_5_ micro-flowers consisted of 3D hierarchical nanosheets with 400–500 nm in diameter have a good crystal phases and a surface area up to 48.6 m^2^ g^−1^. Such 3D porous structure provides abundant catalytic active sites and lead to a superior catalytic activity with degradation rate of 0.208 min^−1^, which is about 60-fold and 4-fold larger than that without the catalysts (W/O) and commercial TiO_2_ (P25) sample, respectively. The photocatalytic reuse tests also show that the performance of Nb_2_O_5_ micro-flowers has a long life and high stability. This present work insights into the design and synthesis of highly active and stable Nb_2_O_5_ photocatalysts for the degradation of dyes.

## Data Availability

The data presented in this study are available on request from the corresponding author.
